# Sex differences in youth with mental health problems in inpatient, outpatient and youth justice settings

**DOI:** 10.1186/s12888-019-2413-z

**Published:** 2020-01-16

**Authors:** Shannon L. Stewart, Elizabeth Thornley, Natalia Lapshina, Patricia Erickson, Evelyn Vingilis, Hayley Hamilton, Nathan Kolla

**Affiliations:** 1 Faculty of Education, Applied Psychology, Western University, 1137 Western Road, Office 1142M, London, Ontario N6G 1G7 Canada; 2 Department of Sociology, University of Toronto, Toronto, Ontario Canada; 3 Schulich School of Medicine, Western University, London, Ontario Canada; 4 Centre for Addiction and Mental Health, Toronto, Ontario Canada

**Keywords:** Youth justice, interRAI, Mental health, Traumatic life experiences, Inpatients and outpatients

## Abstract

**Background:**

Approximately 40–70% of justice-involved youth have untreated mental health problems. There is no current research that directly compares the mental health profiles of youth involved in the justice system to that of inpatients and outpatients. The research reported is significant because it directly compares the needs of these population by use of the same suite of standardized assessment tools.

**Methods:**

The sample consisted of 755 youth aged 16–19 years recruited from youth justice and mental health facilities in Ontario, Canada. Participants completed semi-structured assessment interviews using the interRAI child and youth suite of instruments to assess for internalizing and externalizing concerns as well as exposure to traumatic life events.

**Results:**

Findings indicated that justice-involved youth experienced higher levels of certain types of trauma. Analyses examining sex differences indicated that, controlling for age, males in the youth justice group reported higher cumulative trauma compared to male outpatients but not inpatients. Females in the youth justice group reported experiencing higher cumulative trauma compared to female outpatients and inpatients. In addition, controlling for sex and age, the youth justice group reported lower internalizing symptoms scores than inpatients and outpatients. Finally, males in the youth justice group scored lower than inpatients in externalizing symptoms, whereas females within the youth justice group scored higher in externalizing symptoms compared to inpatients and outpatients.

**Conclusions:**

Results indicated that youth who are involved with the justice system exhibit significant psychosocial issues that represent complex service needs which require unique interventions in order to be addressed appropriately.

## Background

Adolescence is a developmental period characterized by substantial biological, psychological, and social changes. Such changes can lead to risk factors that can increase vulnerability to the development of mental health problems (e.g., changes in mood, conflict with caregivers, identify formation, risky behaviours [1, 2];). In fact, recent research has estimated that 10–25% of all youth meet criteria for a mental health disorder [2–6]. As such, mental health issues are relatively common in the general youth population; however, there is an overall consensus that youth involved in the justice system experience very high rates of mental health issues [4, 7, 8]. Specifically, it is estimated that compared to the 10–25% of all youth that meet criteria for a mental health disorder, approximately 65% of youth in custody have a diagnosable mental health condition [7]. Over the past decade, there has been increased attention on research and practice that would improve the understanding of, and response to, the mental health needs of justice-involved youth [9]. Evidence suggests that youth involved with the justice system have complex mental health needs similar to youth in other service sectors. However, a comprehensive examination of the needs of these complex youth, compared to those in inpatient and outpatient services, has yet to be accomplished [10]. The current study, provides much needed estimates of the prevalence of trauma exposure and mental health problems among male and female youth in these service sectors, further emphasizing the importance of proper identification of these issues through high-quality assessments geared to the prevention of continued offending and adverse long-term sequelae.

### Mental Health needs of justice-involved Youth compared to general population

In Canada, a small number of studies have compared the prevalence rates of mental health issues among youth from the general community and those within the justice system. For example, Ulzen and Hamilton [11] examined the presence or absence of symptoms using the Diagnostic Interview for Children and Adolescents-Revised (DICA-R) in a sample of 49 incarcerated youth and 49 youth from the community. Results indicated that over 85% of incarcerated youth met criteria for at least one DSM-III-R disorder, compared to 30% of youth in the general population. Not surprisingly, the most common diagnoses found within the justice-involved group were those related to disruptive behaviour disorders, such as Oppositional Defiance Disorder (ODD; 45%) and Conduct Disorder (CD; 31%), followed by Alcohol Dependence (39%). In addition, youth within the justice-involved group were more than five times as likely to have one or more disorders (e.g., high level of comorbidity), as compared to the community sample (63 and 12%, respectively).

A more recent study by Gretton and Clift [12] examined the point prevalence rates of specific mental health issues amongst justice-involved youth in British Columbia, Canada. To investigate mental health issues in 145 male and 65 female incarcerated youth, the authors utilized forensic records in tandem with two mental health assessment tools. First, the Massachusetts Youth Screening Instrument Version 2 (MAYSI-2 [13];) was used to screen for mental health issues (e.g., alcohol and drug use, anger/irritability, depression/anxiety, somatic complaints, suicide ideation, thought disturbance, traumatic experiences). Second, provisional psychiatric diagnoses were assessed with the Diagnostic Interview Schedule for Children Version IV (DISC-IV [14];), a structured interview based on the DSM-IV. The three MAYSI-2 subscales for which youth scored above the caution cut-off score were: (1) alcohol and drug misuse (80% of males, 81% of females); (2) anger and irritability (56% of males and 63% of females); (3) depression and anxiety (32% of males and 54% of females). Overall, results using the DISC-IV indicated that 92% of males and 100% of females qualified for at least one diagnosis from the DSM-IV. The most common single diagnosis identified for both males and females was CD (73% of males and 84% of females). When individual substance use disorders were counted as a single category, they were more common than CD (86% of males and 100% of females). Furthermore, anxiety disorders (excluding Posttraumatic Stress Disorder [PTSD]) were common (18% of males and 30% of females), as were mood disorders (6% of males and 7% of females). High rates of comorbidity were also identified, as 73% of males and 88% of females met criteria for at least two separate disorders. Overall, the results from the small number of Canadian epidemiological studies suggest that youth within the Canadian justice system experience a greater occurrence of mental health issues and comorbidity than youth in the general population.

In addition to substance use, anxiety, and depression, rates of exposure to traumatic events are also high in justice-involved youth. The majority of North Americans will experience at least one traumatic event prior to 18 years of age [15]. Based on epidemiological studies, it has been estimated that 92.5% of justice-involved youth have experienced at least one trauma, whereas 84.0% have experienced more than one trauma (mean: 14.6, median: 6 number of traumatic incidents) [16]. Examples included physical (35.3%) or sexual abuse (4.4%), witnessing domestic violence (74.1%), being threatened with a weapon (58.4%) and other traumatic experiences [16].

Exposure to traumatic events varies by sex and is associated with a variety of negative long-term outcomes [17] and can lead to the development of PTSD. In a sample of 252 adolescents admitted to two juvenile detention centers in Maine, USA, over 70% of females were subjected to some form of abuse, compared to almost 45% of males [18]. On the other hand, in a sample of 898 arrested and newly detained youth in Illinois, USA, significantly more males (93.2%) reported at least one traumatic experience compared to females (84.0%) [16]. Not surprisingly, females report significantly higher rates of sexual abuse compared to males [16, 18]. The rates of physical abuse were similar (females: 35%, males: 28%), whereas females experienced statistically higher rates of emotional abuse (females: 50%, males: 27%) [18]; however, significantly more males than females reported being in a “bad accident” [16]. The overall prevalence of PTSD in the general population is 3.5% [4], whereas in detained youth it is 11.2% [18]. As with traumatic experiences, the rates of PTSD vary by sex, although findings are not consistent. Gretton and Clift [12] identified point prevalence rates of PTSD in a justice-involved sample as being 1.7% of males and 13.0% of females meeting the criteria for a provisional diagnosis. Similarly, Abrantes, Hoffmann and Anton reported higher rates of PTSD in females (35%) compared to males (15%) [18]. On the other hand, Abram and colleagues [16] reported no significant sex differences in PTSD diagnosis.

Therefore, youth involved in the justice system represent a particularly vulnerable population. Risk factors, such as learning difficulties, comorbid emotional and behavioural problems, substance abuse, exposure to trauma, place these youth at risk for developing serious and pervasive mental health problems [2].

### Mental Health needs of justice-involved youths compared to Mental Health service sector

Although much research has compared the mental health needs of justice-involved youth to overall community samples, there is significantly less research that compares the mental health needs of youth across multiple service sectors (e.g., inpatient and outpatient mental health services). Research that has addressed these issues has highlighted the significant overlap between the mental health needs of youth within the juvenile justice system and those involved with mental health services [19–22]. Some of this overlap has been investigated and attributed to youths involved in both service sectors. For example, Rosenblatt, Rosenblatt, and Biggs [23] examined data from 4924 youth involved in both the public mental health and juvenile justice systems. They found that 20% of youth receiving mental health services had recent arrest records and 30% of youth arrested received mental health services. They further compared a subsample of 94 mental health service users with arrests to 94 mental health service users without arrests. Not surprisingly, compared to the non-arrest group, they found youth with histories of arrests had a higher frequency of CD/ODD. Furthermore, youth with arrest histories had higher Externalizing and Total Problem Scale scores as well as more functional impairment, as measured by the Child Behavior Checklist [24]. However, youth receiving mental health services with an arrest history were less likely to have a diagnosis of an anxiety disorder compared to youth involved in mental health services with no arrest record. This may be because participants in this study were assigned a primary DSM-IV diagnosis. Therefore, youth with an arrest record may have received a different diagnosis that reflected their current needs associated with externalizing concerns (e.g., ODD/CD) although they may also have met criteria for an internalizing disorder. In addition, no significant differences between groups were found for mood disorders.

In terms of traumatic experiences, the prevalence of maltreatment in youth in juvenile justice sector was similar to mental health sector (77.6 and 75.1% respectively), and lower than in alcohol/drug users (86.3%) and child welfare youth (85.3%). Controlling for sex, race/ethnicity, and age, youth in child welfare were significantly more likely to report multiple types of maltreatment than those in mental health or juvenile justice [25].

Various studies have found that extralegal factors, including individual and social or environmental characteristics influence how youth engage in various service settings such as race, ethnicity, mental health, and trauma histories [10]. For example, studies have found that many youths with mental health needs are at a disproportionate risk of being directed to the juvenile justice system [26]. Specifically, youth with CD, ODD, and substance use issues are commonly directed towards a youth justice pathway [10]. Recent research has indicated that males and females differ on their trajectories towards the juvenile justice sector [27]. For example, for youth in the community, more males had an onset of antisocial behavior in childhood (ratio 10:1) than females. Conversely, the ratio dropped to 1.5:1 for males and females when the onset of antisocial behavior occurred in adolescence [28]. However, for youth involved in the justice system, there is evidence that the co-occurrence of both internalizing and externalizing issues is more common in females [29], placing them at greater risk for custody and detention involvement, greater complexity of need as well as recidivism. As such, rates of externalizing behaviour between community and justice-involved youth may be vastly different and thus it is imperative to view the mental health needs of youth within the justice system in consideration of these differences. Social and environmental characteristics that have been found to influence youth involvement in the justice system include family conflict, lack of available services, and prior service usage [10]. As such, relevant research has highlighted high levels of mental health needs in the youth justice system and further suggest that these needs may be unique compared to youth receiving mental health services who are not involved in the justice system and these differences may also vary by sex.

To our knowledge, there is no research directly comparing the profiles and histories of youth involved in the justice system, youth receiving inpatient mental health services, and youth receiving outpatient mental health services. In addition, the current study is significant because it describes mental health and related outcomes of youth across these various settings and reports the prevalence of additional risk factors for these youth, such as various types of traumatic experiences. The research clearly delineates differences that exist between the three groups of youth in terms of traumatic life events and mental health needs and helps guide knowledge for service improvements.

#### Hypotheses

The aim of the current study is to directly examine and compare the mental health similarities and differences of youth across those three service settings. While this study is exploratory in nature, certain predictions are forwarded. Specifically, based on previous literature, it is predicted that youth within the justice system, compared to those within outpatient and inpatient mental health services, will experience: (1) higher rates of traumatic events; (2) more externalizing problems; (3) fewer internalizing problems. With respect to sex differences, it is expected that the difference between youth justice, inpatient and outpatient groups will be moderated by sex, such that the differences will be more pronounced in females relative to males.

## Method

### Participants

The sample consisted of 755 youth (*M age =* 16.76, *SD* = .81); of those, 47.4% identified as male (see Table 1). Almost 8 % (7.7%) identified as Indigenous (example: First Nations, Metis, Inuit). Inpatient and outpatient groups were referred from 22 mental health agencies and the justice group was from 10 secure custody sites across the Province of Ontario. The three samples included all youth who were admitted to inpatient, outpatient, or youth custody/detention facilities in Ontario, between 16 and 19 years of age. The majority of participants (*n* = 590; 78.1%) were from outpatient services. Approximately 10% (*n* = 75; 9.9%) were inpatients while almost 12% (*n* = 90; 11.9%) were youth in the justice system. Ethnic differences were not reported due to ethical concerns around the characteristics of small samples (in order to protect participant confidentiality). Consent for inpatient and outpatient participants differed from those within the justice system. For the inpatient and outpatient participants, both caregivers and youth provided written consent as part of standard care at the mental health facility. Within the justice system, youth were considered competent and able to provide consent only if they were deemed capable of understanding the purpose of the research, foreseeable risks, potential benefits and the consequences of the research. If the youth was determined to have diminished capacity (e.g., cognitively impaired) they were not included in the study. All competent youth were required to be over 16 years of age, and admitted to either a youth justice facility, inpatient or outpatient unit. Only initial assessments were utilized to prevent duplication of assessments. Furthermore, a unique case record number was utilized for each youth within either the youth justice, inpatient or outpatient service sector.

**Table 1:** Sample Demographics by Case Type (*N* = 755)

Variable	Case Type		
	Youth Justice Group *N* = 90	Inpatients *N* = 75	Outpatients *N* = 590
Age (*M, SD*)	17.24 (.89)	16.83 (.78)	16.68 (.78)
Sex (%)			
Male	76.7	50.7	42.5
Female	23.3	49.3	57.5

### Assessment instruments

Child and Youth interRAI instruments [30–32]- Stewart, Hirdes, McKnight et al., 2018 [33]; are comprehensive assessment systems that require approximately 1 hour for completion. Each instrument is based on a semi-structured interview of individual needs (e.g., assessment of psychiatric, substance use, social, environmental, and medical issues, with emphasis on individual functioning) with applications to support decisions related to care planning and outcome measurement. Multiple reliability (e.g., inter-rater) and validity studies (e.g., construct validation, concurrent validity, predictive validity, internal consistency) have demonstrated strong psychometric properties for interRAI instruments in adult and geriatric samples [34–37] and in children/youth samples [38–43], with acceptable or higher average levels of inter-rater reliabilities [44]. Each assessment instrument within the interRAI mental health suite shares similar items, scales, and CAPs that have been validated across multiple service sectors. For youth justice, there is additional information collected including age of first criminal involvement, charges and convictions, and family history of criminal offences. Additional items related to control interventions (e.g., restraint use), discharge planning and resources available upon discharge were included within the inpatient assessment. InterRAI instruments have been utilized for a wide variety of children and youth with different presenting concerns [45–52], including youth in conflict with the law [53]. For the purposes of this study, only information that was common across all instruments were utilized.

The assessment system includes a data collection form, a user manual, triggers, and Collaborative Action Plans (CAPs). The “trigger” items indicate the presence of imminent risk of problems that affect the youth. These trigger items comprise algorithms that flag youth with potential problems in need of further clinical review (e.g., self-harm, substance use) and where appropriate, activate a CAP. Each CAP is accompanied by the reason for the identification of the clinical problem, specifications in trigger algorithms used to flag youth with the potential issue, a background of the current best practice evidence related to the clinical problems, and questions to probe for as part of a more detailed clinical review.

The *interRAI Youth Justice Custodial Facilities* (YJCF; in pilot) [33] instrument contains 416 items, with subsections specific to this population. In particular, the assessment includes items related to criminal involvement, and triggers for such CAPs as risk of continued offending, rationalizations for antisocial choices, and fire setting [40].

The *interRAI Child and Youth Mental Health* (ChYMH) [30] assessment instrument consists of 425 items. Similar to the interRAI YJCF, it measures specific sections and items that trigger such CAPs as attachment, caregiver distress, informal support, life skills, and parenting. Assessments were conducted in person at the time of initial admission to the inpatient or outpatient mental health facilities. Items and scales that are consistent across all instruments were included in analysis for comparison purposes.

### Measures

The outcome measures utilized in this study are part of the interRAI assessment and included externalizing and internalizing symptoms and traumatic life events. The *Internalizing Scale* measures the frequency and severity of internalizing symptoms (i.e., emotional distress/disturbance). The scale consists of three factors: anhedonia, anxiety, and depression. Three items assess anxiety, such as repetitive anxious complaints/concerns, unrealistic fears, and episodes of panic. Four items assess anhedonia: lack of motivation, anhedonia, withdrawal from activities of interest, and decreased energy. Finally, four items assess depression: made negative comments, self-deprecation, expressions of guilt/shame, and expressions of hopelessness. Item response options range from 0 – *not present* to 4- *exhibited daily in last 3 days, 3 or more episodes or continuously*. Scores were summed, with a range 0 to 44, where higher scores indicate higher levels of internalizing symptoms (Cronbach’s α = .87).

The *Externalizing Scale* measures the frequency of externalizing symptoms: i.e., behavioural disturbance). The scale consists of 12 items that belong to two factors: proactive aggression and reactive aggression. The proactive aggression items include stealing, elopement attempts/threats, bullying peers, preoccupation of violence, violence to others, intimidation of others or threatened violence, and violent ideation. The reactive aggression factor includes impulsivity, verbal abuse, outburst of anger, and defiant behaviour. Five items are measured using the 0 to 4 scale, while seven items are measured using a 0 to 5 scale. To obtain a total score for the Externalizing Scale, item scores were recoded such as any score of 0 remained as zero, and any score ranging from 1 to 5 was recoded to 1. Scores range between 0 to 12, with higher scores indicating higher levels of externalizing symptoms (Cronbach’s α = .87).

*Traumatic Life Events* were assessed with 14 questions that address a variety of traumatic events experienced by a youth, e.g., death of a parent or primary caregiver, witness of severe accident, being a victim of physical or sexual assault or abuse. Response options ranged from 0*- never,* 1*- more than 1 year ago, 2–31 days-1 year ago, 3–8-30 days ago, 4–4-7 days ago, and 5- present within the last 3 days.* Due to low counts of recent traumatic life events, the responses were dichotomized into 0 - *never* and 1 - *more than 1 year ago to in the last 3 days*. The above-mentioned forms of traumatic life events responses were further summed resulting in an interval *Cumulative Trauma* variable that ranged from 0 to 14, where higher values indicated more forms of trauma experienced by a youth in the past. Both the Child and Youth Mental Health instrument [30] and the Youth Justice Custodial Facilities Instrument [33](manuscript under review) have been validated in English.

### Procedure

InterRAI assessors received a standardized two-day training in the context-specific assessment tool. After the training, booster sessions and access to webinars were available to support assessment, coding and uptake of CAPS.

Assessments in inpatient and outpatient facilities were administered as part of standard clinical practice utilizing the interRAI Child and Youth Mental Health Assessment (ChYMH) [30]. Every inpatient or outpatient received a unique identifier (CRN) that was used to track his or her clinical history since the first admission. In the case where a youth had multiple admissions during the study period, data from the first admission were kept in the data set; all follow-up data were discarded, to avoid duplication.

In the Youth Justice facilities, assessors completed the Youth Justice Custodial Assessment (YJCF; under review) [33] with consenting youth within 72 h after admission, or as soon as practicable. Specifically, assessments were generally completed within 3 days of admission to a detention facility, inpatient or outpatient service. However, if youth were agitated during the 72-h admission period to the inpatient or custody/detention facility, the assessment was conducted once the youth had time to settle. A quarter (25.2%) of youth in custody or detention agreed to participate in the study which is consistent with other research with youth justice participants. Youth may have been wary of involvement in research to avoid potential negative consequences after a disclosure of information that may be considered as sensitive (e.g., disclosure of anxiety, involvement with other illegal activities).

Each interRAI instrument took approximately 1 hour to complete. Response sets and items utilized in this study were identical. Psychometrics of the interRAI scales have been found to be consistent across samples and service sectors [34–42, 44].

The data were collected between October 2012 and November 2016 using the interRAI Child and Youth Mental Health (ChYMH) for patient groups and interRAI Youth Justice Custodial Facilities (YJCF) instruments for the youth in custodial facilities between November 2014 and November 2016. Both instruments have comparable structure and scales and are standardized instruments that are based on a semi-structured interview format. Trained clinicians (e.g., social workers, child and youth works, justice workers, psychologists, nurses) working within the agencies and facilities conducted the semi-structured interviews using a paper or online format. In the case of paper format, after the assessment a clinician transferred the data into an online software. Every assessment has to be completed in its entirety in order to be successfully submitted and scored using the interRAI platform; consequently, there were no missing cases in the data set. The data were stored in electronic format on an interRAI server, and then transferred and stored securely in University of Western Ontario computers with no internet access.

### Analytic strategy

The results were analyzed using IBM SPSS Statistics package, version 25. To address the departure from normality, Spearman’s bivariate correlations were utilized to examine the relationships among age and outcome variables (cumulative trauma, externalizing, internalizing symptoms).

Frequency analyses were conducted to examine sex differences and the prevalence of traumatic life events depending on case type and the overall prevalence of trauma in the sample. Binary logistic regression analyses examined the prevalence of traumatic life events as a function of case type, adjusting for sex and age. The binary logistic regression assumptions were satisfied. Odds ratios (*OR*) derived from binary logistic regression analyses were utilized to compare case type and sex differences in traumatic life events, controlling for age.

Generalized linear models (GLM) were utilized to examine age-adjusted case type and sex differences in cumulative trauma, externalizing and internalizing symptoms. The GLM with a gamma error distribution and robust standard error estimation was chosen to address the positively skewed and light-tailed distribution of standardized residuals with some outlying values [54, 55]. Analyses probed for significant interaction between case type and sex. The models included two main effects (case type: YJ, outpatients, inpatients; and sex: male, female), a case type × sex interaction, and age as a covariate. In the models, male and youth justice group served as reference categories for sex and case type respectively. To follow up significant interactions, post- hoc analyses were conducted to examine case type differences separately for males and females.

Effect sizes were estimated by Nagelkerke *R*^*2*^ for binary logistic regression and Zheng and Agresti’s *R*^*2*^ [56], which is a squared correlation between the observed and the predicted response. All statistical tests were two-tailed. The significance level was set at alpha .05, which corresponded to 95% confidence intervals in logistic regression analyses. Bonferroni corrections were utilized to account for multiple comparisons, by dividing the unadjusted *p-*value by the number of comparisons and then compare it with alpha (.05).

## Results

### Preliminary analyses

#### Sample descriptive statistics

Demographics specific to each sample in the study are listed in Table 1. The youth justice group included participants who were older and had higher proportion of males than the patient groups.

#### Bivariate relationships among continuous predictors and outcomes

Table 2 lists descriptive statistics (means and standard deviations) for age, cumulative trauma, externalizing and internalizing symptoms, and Spearman bivariate correlations. All three outcome measures were positively skewed, had relatively wide standard deviations, and positively related to each other. Age was weakly positively related to cumulative trauma (*r*_*s*_ (755) = .09, *p* = .014) but not externalizing (*r*_*s*_ (755) = −.07, *p* = .050) or internalizing symptoms (*r*_*s*_ (755) = −.06, *p* = .079).

**Table 2:** Descriptive Statistics and Spearman Bivariate Correlations among Continuous Predictors and Outcome Measures (*N* = 755)

Variable	*M (SD)*	1	2	3	4
1. Age	16.76 (.81)	–			
2. Cumulative trauma	3.28 (2.92)	.09*	–		
3. Externalizing problems	4.16 (3.52)	−.07	.42***	–	
4. Internalizing problems	11.09 (9.25)	−.06	.14***	.15***	–

* *p* < .05, *** *p* < .001

### Prevalence of traumatic life events by case type

To test the hypothesis regarding higher rates of traumatic events in youth within the justice system compared to mental health outpatients and inpatients, frequencies of traumatic life events were examined first. Next, binary logistic regression analyses were utilized to examine the prevalence of traumatic life events depending on case type after adjusting for age and sex. Finally, age-adjusted differences in cumulative trauma depending on case type and sex were investigated using Generalized Linear Modelling.

#### Frequencies of traumatic life events

Table 3 provides frequencies of traumatic life events depending on case type and a total summary of various traumatic life events. In the youth justice group, the five most prevalent traumatic life events were “failed or dropped out of an education program” (64.4%), living in a violent neighborhood (60.0%), death in the family (56.7%), being a victim of bullying (54.4%), and a victim of emotional abuse (52.2%). Inpatients experienced bullying (56.0%), emotional abuse (45.3%), witnessed domestic violence (40.0%), changed a custodian (38.7%), and physical abuse (36.0%) as the top five traumatic life experiences. In the outpatients, the five most prevalent traumatic experiences included being a victim of bullying (51.9%), emotional abuse (34.9%), death in the family (32.9%), witnessing domestic violence (24.1%), and being a victim of physical abuse (22.2%). Likewise, in the total sample, the five most prevalent traumatic life experiences included being a victim of bullying (52.6%), emotional abuse (38.0%), death in family (34.7%), witnessing domestic violence (28.3%), and being a victim of physical abuse (26.8%).

**Table 3:** Frequencies of Traumatic Life Events by Case Type (N = 755)

Traumatic Life Event	Youth Justice *n* (%)	Inpatients *n* (%)	Outpatients *n* (%)	Total *n* (%)
Victim of sexual violence	14 (15.6)	19 (25.3)	96 (16.3)	129 (17.1)
Victim of physical abuse	44 (48.9)	27 (36.0)	131 (22.2)	202 (26.8)
Victim of emotional abuse	47 (52.2)	34 (45.3)	206 (34.9)	287 (38.0)
Parental death	24 (26.7)	26 (21.3)	77 (13.1)	117 (15.5)
Custodian change	41 (45.6)	29 (38.7)	95 (16.1)	165 (21.9)
Death in family	51 (56.7)	17 (22.7)	194 (32.9)	262 (34.7)
Parental addiction	39 (43.3)	22 (29.3)	111 (18.8)	172 (22.8)
Victim of bullying	49 (54.4)	42 (56.0)	306 (51.9)	397 (52.6)
Parental abandonment	42 (46.7)	21 (28.0)	81 (13.7)	144 (19.1)
Witness of domestic violence	42 (46.7)	30 (40.0)	142 (24.1)	214 (28.3)
Violent neighborhood	54 (60.0)	11 (14.7)	43 (7.3)	108 (14.3)
Victim of crime	34 (37.8)	7 (9.3)	29 (4.9)	70 (9.3)
Serious accident or physical impairment	19 (21.1)	11 (14.7)	56 (9.5)	86 (11.4)
Failed or dropped out of education program	58 (64.4)	21 (28.0)	113 (19.2)	192 (25.4)

Traumatic life events coded so that 0 = never experienced and 1 = experienced in last 3 days- more than 1 year ago

#### Age and sex-adjusted prevalence of traumatic life events

Next, binary logistic regression analyses were conducted to examine differences in traumatic life events as a function of case type, controlling for sex and age. In the models, male and youth justice group served as reference categories for sex and case type respectively. Table 4 summarizes these findings. Age was not related to any traumatic life event. Sex was related to being a victim of sexual violence (*OR* = 5.06, 95% CI [3.13, 8.19]), parental death (*OR* = 1.89, 95% CI [1.23, 2.90]), and being a victim of emotional abuse (*OR* = 1.63, 95% CI [1.19, 2.22]). Specifically, regardless of case type, females had five times the odds of experiencing sexual violence, almost two times the odds of experiencing parental death, and about one and a half times the odds of experiencing emotional abuse than males.

**Table 4:** Age and Sex Adjusted Logistic Regression Analyses Predicting Traumatic Life Events as a Function of Case Type

	Coefficients			95% *OR CI*		
	*B (SE)*	*p*	*OR*	Lower bound	Upper bound	Nagelkerke *R*^*2*^
Victim of sexual violence						.123
Female (vs. male)	1.62 (.25)	<.001	5.06	3.13	8.19	
Case type						
Inpatient (vs. YJ)	.26 (.42)	.545	1.29	.57	2.94	
Outpatient (vs. YJ)	−.47 (.34)	.169	.63	.32	1.22	
Victim of physical abuse						.063
Case type						
Inpatient (vs. YJ)	−.48 (.33)	.141	.62	.33	1.17	
Outpatient (vs. YJ)	−1.14 (.25)	<.001	.32	.20	.52	
Parental death						.053
Female (vs. male)	.64 (.22)	.004	1.89	1.23	2.90	
Case type						
Inpatient (vs. YJ)	−.56 (.38)	.148	.57	.27	1.22	
Outpatient (vs. YJ)	−1.23 (.29)	<.001	.29	.17	.52	
Custodian change						.095
Case type						
Inpatient (vs. YJ)	−.29 (.33)	.367	.75	.40	1.41	
Outpatient (vs. YJ)	−1.49 (.26)	<.001	.23	.14	.37	
Death in family						.047
Case type						
Inpatient (vs. YJ)	−1.42 (.35)	<.001	.24	.12	.49	
Outpatient (vs. YJ)	−.87 (.24)	<.001	.42	.26	.67	
Victim of emotional abuse						.038
Female (vs. male)	.49 (.16)	.002	1.63	1.19	2.22	
Case type						
Inpatient (vs. YJ)	−.41 (.32)	.200	.66	.35	1.24	
Outpatient (vs. YJ)	−.89 (.24)	<.001	.41	.25	.66	
Parental addiction						.054
Case type						
Inpatient (vs. YJ)	−.65 (.34)	.055	.52	.27	1.02	
Outpatient (vs. YJ)	−1.24 (.25)	<.001	.29	.18	.48	
Victim of bullying						.005
Case type						
Inpatient (vs. YJ)	.02 (.32)	.943	1.02	.55	1.91	
Outpatient (vs. YJ)	−.16 (.24)	.512	.86	.54	1.37	
Parental abandonment						.105
Case type						
Inpatient (vs. YJ)	−.85 (.34)	.013	.43	.22	.84	
Outpatient (vs. YJ)	−1.75 (.26)	<.001	.17	.10	.29	
Witness of domestic violence						.046
Case type						
Inpatient (vs. YJ)	−.32 (.32)	.318	.72	.39	1.36	
Outpatient (vs. YJ)	−1.08 (.25)	<.001	.34	.21	.55	
Violent neighborhood						.283
Case type						
Inpatient (vs. YJ)	−2.23 (.40)	<.001	.11	.05	.24	
Outpatient (vs. YJ)	−3.04 (.30)	<.001	.05	.03	.09	
Victim of crime						.204
Case type						
Inpatient (vs. YJ)	−1.58 (.46)	.001	.21	.08	.51	
Outpatient (vs. YJ)	−2.19 (.31)	<.001	.11	.06	.21	
Serious accident or physical impairment						.036
Case type						
Inpatient (vs. YJ)	−.35 (.43)	.409	.70	.31	1.62	
Outpatient (vs. YJ)	−.82 (.31)	.009	.44	.24	.82	
Failed or dropped out of education program						.145
Case type						
Inpatient (vs. YJ)	−1.43 (.34)	<.001	.24	.12	.47	
Outpatient (vs. YJ)	−1.87 (.26)	<.001	.15	.09	.25	

Traumatic life events coded so that 0 = never experienced and 1 = experienced in last 3 days- more than 1 year ago

Covariates that were not statistically significant (*p* > .05) were omitted from the table results

Controlling for age and sex, there were no significant differences between youth justice, inpatients, and outpatients in the likelihood of experiencing sexual violence (inpatients vs. YJ: *OR* = 1.29, 95% CI [.57, 2.94]; outpatients vs. YJ: *OR* = .63, 95% CI [.32, 1.22]) and bullying (inpatients vs. YJ: *OR* = 1.02, 95% CI [.55, 1.91]; outpatients vs. YJ: *OR* = .86, 95% CI [.54, 1.37]).

Compared to the youth justice group, outpatients were less likely to experience physical abuse (*OR* = .32, 95% CI [.20, .52]), parental death (*OR* = .29, 95% CI [.17, .52]), custodian change (*OR* = .23, 95% CI [.14, .37]), emotional abuse (*OR* = .41, 95% CI [.25, .66]), parental addiction (*OR* = .29, 95% CI [.18, .48]), serious accident or physical impairment (*OR* = .44, 95% CI [.24, .82]), or witness domestic violence (*OR* = .34, 95% CI [.21, .55]), after adjusting for sex and age. However, as seen in Table 4, the youth justice group and inpatients did not differ in the likelihood of experiencing these traumatic events.

Finally, both patient groups were less likely to experience death in family (inpatients: *OR* = .24, 95% CI [.12, .49]; outpatients: *OR* = .42, 95% CI [.26, .67]), parental abandonment (inpatients: *OR* = .43, 95% CI [.22, .84]; outpatients: *OR* = .17, 95% CI [.10, .29]), live in a violent neighbourhood (inpatients: *OR* = .11, 95% CI [.05, .24]; outpatients: *OR* = .05, 95% CI [.03, .09]), being a crime victim (inpatients: *OR* = .21, 95% CI [.08, .51]; outpatients: *OR* = .11, 95% CI [.06, .21]), or failed or dropped out of education program (inpatients: *OR* = .24, 95% CI [.12, .47]; outpatients: *OR* = .15, 95% CI [.09, .25]) than the youth justice group, controlling for sex and age.^1^

#### Cumulative trauma

**Fig. 1:**
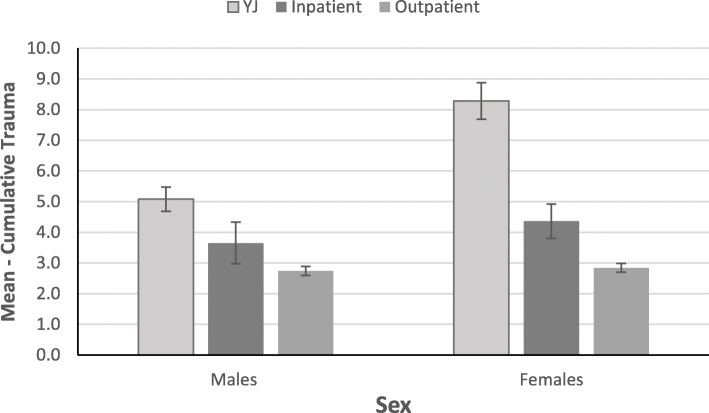
Age-Adjusted Case type Differences in Mean Cumulative Trauma by Sex

Gamma GLM was utilized to investigate the age-adjusted differences in cumulative trauma as a function of case type and sex. First, analyses probed for an interaction between case type and sex. The interaction was significant, Wald *χ*^*2*^(1) = 15.28, *p* < .001 (full model Likelihood Ratio *χ*^*2*^(6) = 21.80, *p* = .001, Zheng and Agresti *R*^*2*^ = .147). To further examine the significant interaction, case type differences in cumulative trauma were examined as a function of sex, after adjusting for age. Figure 1 depicts the interaction.

The difference was significant in males (Wald *χ*^*2*^(2) *=* 24.70, *p* < .001, Zheng and Agresti *R*^*2*^ *=* .101), where males in the youth justice group reported higher cumulative trauma experiencing almost five traumatic life events on average (*M* = 4.97, *SE* = .41) compared to male outpatients (*M* = 2.77, *SE* = .16; *p* < .001). The difference between the youth justice group and inpatients was not significant (inpatients: *M* = 1.35, *SE* = .67, *p* = .131). Likewise, male inpatients and outpatients did not differ from each other in terms of cumulative trauma (*p* = .402).

The difference in cumulative trauma was significant in females (Wald *χ*^*2*^(2) *=* 66.19, *p* < .001, Zheng and Agresti *R*^*2*^ *=* .183). Pairwise comparisons with Bonferroni correction revealed that females in the youth justice group reported experiencing eight types of trauma on average (*M* = 8.27, *SE* = .69) and scored higher compared to female outpatients (*M* = 2.84, *SE* = .14; *p* < .001) and inpatients (*M* = 4.36, *SE* = .51, *p* < .001). Female inpatients scored higher in cumulative trauma than outpatients (*p* = .013).

### Internalizing and externalizing symptoms

To test the hypothesis regarding more pronounced differences in externalizing and internalizing symptoms in females relative to males, analyses probed for an interaction between case type and sex using GLM. As in the case with cumulative trauma, the models included two main effects (case type: YJ, outpatients, inpatients; and sex: male, female), a case × sex interaction, and age as a covariate. The case × sex interaction was significant in the case of externalizing symptoms, Wald *χ*^*2*^(2) = 13.51, *p* = .001 (full model Likelihood Ratio *χ*^*2*^ (6) = 27.16, *p* < .001, Zheng and Agresti *R*^*2*^ = .138). In the case of internalizing symptoms, the interaction was not significant, Wald *χ*^*2*^(2) = 1.15, *p* = .562; therefore, the models omitted the interaction and included the main effects of case type and sex.

#### Externalizing symptoms

**Fig. 2:**
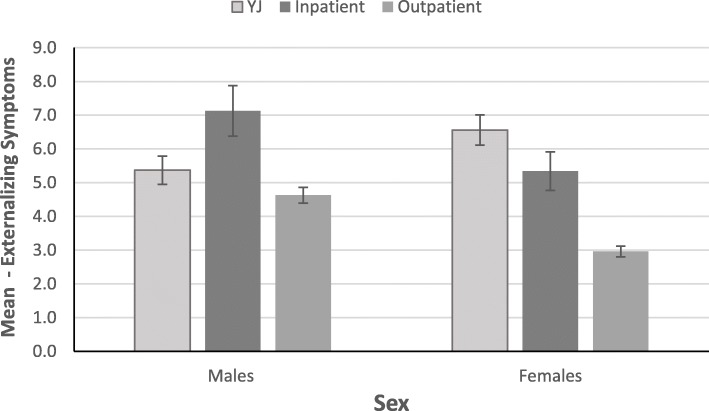
Age-Adjusted Case Type in Mean Externalizing Symptoms by Sex

To further examine the significant interaction, case type differences in externalizing symptoms were examined separately in males and females, after adjusting for age. Figure 2 depicts the interaction.

The difference was significant in males (Wald *χ*^*2*^(2) *=* 27.84, *p* < .001, Zheng and Agresti *R*^*2*^ *=* .058). Pairwise comparisons with Bonferroni correction revealed that males in the youth justice group scored lower in externalizing symptoms than male inpatients (youth justice: *M* = 5.54, *SE* = .42; inpatients: *M* = 7.12, *SE* = .43, *p* = .023); however, they did not differ from male outpatients (*M* = 4.54, *SE* = .24; *p* = .134). Male inpatients scored significantly higher than outpatients (*p* < .001).

The difference in externalizing symptoms was significant in females (Wald *χ*^*2*^(2) *=* 45.75, *p* < .001, Zheng and Agresti *R*^*2*^ *=* .130). Pairwise comparisons with Bonferroni correction revealed that females in the youth justice group scored four points higher in externalizing symptoms than female outpatients (youth justice: *M* = 7.17, *SE* = .74; outpatients: *M* = 2.95, *SE* = .16; *p* < .001). However, females in the youth justice and inpatients did not differ from each other in externalizing symptoms (inpatients: *M* = 5.45, *SE* = .58, *p* = .203). Female inpatients scored higher in externalizing symptoms than outpatients (*p* < .001).

#### Internalizing symptoms

The model was significant, Likelihood Ratio *χ*^*2*^(4) = 33.89, *p* < .001, Zheng and Agresti *R*^*2*^ *=* .070. Age was not related to internalizing symptoms, Wald *χ*^*2*^(1) = .259, *p =* .611. The main effect of sex was significant, Wald *χ*^*2*^(1) = 13.91, *p* < .001, wherein as expected, females (*M* = 10.49, *SE* = .57) scored higher than males (*M* = 7.95, *SE* = .52) in internalizing problems. After controlling for sex differences, the case type differences were significant, Wald *χ*^*2*^(2) = 48.93, *p* < .001. Pairwise comparisons with Bonferroni correction revealed that the youth justice group (*M* = 5.66, *SE* = .78) reported lower internalizing symptoms scores than inpatients (*M* = 9.98, *SE* = .90, *p* = .001) and outpatients (*M* = 12.01, *SE* = .39, *p* < .001), with no differences between the patient groups (*p* = .123).

## Discussion

The current study investigated the mental health similarities and differences of youth across three service settings: (1) youth in justice, (2) youth receiving inpatient mental health services, and (3) youth receiving outpatient mental health services. The groups were compared directly for their rates of internalizing and externalizing symptoms as well as exposure to traumatic life events.

### Trauma

In the sample, traumatic life events varied as a function of case type. Of the fourteen types of traumatic events investigated, only two types – having been a victim of sexual violence or a victim of bullying – did not vary significantly by case type. It was hypothesized that the youth justice group would have high rates of exposure to traumatic life events. Consistent with this hypothesis, trauma rates were found to be higher for youth in justice, particularly when compared to youth receiving outpatient mental health services. Compared to the outpatient mental health group, the justice-involved youth had significantly higher rates of exposure to seven of the eleven trauma types measured: physical abuse, emotional abuse, parental addiction, parental death, change of legal custodian, witnessing domestic violence, and being in a serious accident or having physical impairment. Comparatively, youth in justice and youth receiving inpatient mental health care were found to have relatively similar rates of trauma exposure, drawing further attention to the need for intensive mental health supports for justice-involved youth. However, it is also possible that youth are differentially placed into either youth justice or inpatient services depending on other factors (e.g., race, ethnicity, gender).

Justice-involved youth, compared to both patient groups, had significantly higher rates of exposure to five potentially traumatic events: parental abandonment, death in family, failing educational program, being a victim of crime, and living in a violent community. This finding is also consistent with previous literature [16, 57]; however, the current study was unique in its ability to directly compare youth in justice to youth in inpatient and outpatient mental health programs. The interRAI Child and Youth suite provides an opportunity to compare and contrast sub-groups of vulnerable children and youth to foster integrated care while facilitating continuity of care across service sectors utilizing a common assessment-to-intervention system [58]. As such, unique patterns of trauma exposure were identified for each of the three groups. Interestingly, the youth justice group experienced parental abandonment more often than both patient groups, which is in line with previous research that has investigated pathways of service for justice-involved youth. Specifically, it has been reported that youth who are in contact with law enforcement are also perceived to have less parental support and involvement and are more likely to be placed in a correctional facility as opposed to mental health services [59]. It should be noted that there were no differences between justice-involved youth and patient groups in experiencing sexual violence or bullying on other types of trauma in this study.

Additionally, male youth justice participants reported higher cumulative trauma compared to male outpatients but did not differ from inpatients. Likewise, female youth justice participants reported experiencing eight types of trauma on average, which was significantly higher than female outpatients and inpatients. These results were in line with hypothesis about more pronounced differences in females compared to males. Taken together, the differences in traumatic experiences reported above indicates that the youth justice population was highly affected by traumatic life experiences, and that this is especially true for females involved in the justice system who may present with a particularly complex trauma history. One of the reasons why female youth justice participants scored the highest in cumulative trauma might be due to complex developmental trauma. Indeed, girls in youth justice system tend to be sexually abused and have experienced high degrees of poly-victimization [60, 61]. The relationship between youth in the justice system and exposure to traumatic events appears to be cyclical in which exposure to traumatic life events places youth at risk for criminal involvement. Involvement in the criminal justice system itself places youth at further risk for trauma exposure, which may further exacerbate mental health and legal outcomes [62]. Although the relationship between trauma exposure and justice involvement has been well established [63], the mechanisms underlying this relationship remain uncertain [64, 65]. Previous research efforts have highlighted the potential role of posttraumatic symptoms as a mediator between exposure to violence and self-reported delinquent behaviours [66]. For example, in a sample of detained males, posttraumatic symptoms have been positively associated with the number of past year arrests, past year delinquency severity, number of lifetime arrests but not lifetime delinquency severity, after controlling for age and ethnicity [67]. As such, intervention to address trauma-related symptomatology may be beneficial not only for the mental health of justice-involved youth but also contribute to more favorable legal outcomes, such as decreased rates of recidivism.

The findings of the current study are consistent with previous literature that emphasizes the need to understand youth justice involvement from a developmental psychopathology lens, especially within high risk children, given that behavioral problems are associated with greater probability of incarceration, while emotional problems are associated with greater chance of being sent to residential treatment facilities [59]. The high rates of trauma exposure prevalent amongst justice-involved youth in the current study further emphasize the importance of interventions to prevent long-term sequelae and continued involvement in the justice system. The current study highlights that youth who are involved with the justice system often exhibit significant psychosocial issues that represent complex service needs which require unique interventions in order to be addressed appropriately. As such, it also indicates the importance of further research regarding the effectiveness and implementation of trauma-informed systems for youth involved with the justice system to both better address the impact of trauma on youth involvement in the criminal justice system and to promote successful care for youth with complex mental health needs.

### Internalizing and externalizing symptoms

Based on previous research, analyses probed for interactions between case type and sex in predicting externalizing and internalizing problems. Internalizing issues were examined by use of the Internalizing Scale which included items related to anhedonia, anxiety and depression. The relationship between case type and internalizing symptoms did not depend on sex, which was not in line with the hypothesis. Namely, regardless of case type, females reported higher internalizing symptoms than males. Controlling for sex differences, the justice-involved group was found to report lower anxiety, anhedonia and depression than both inpatient and outpatient groups. No differences were reported between the two patient groups. This is consistent with previous findings in which youth involved in the justice system exhibited lower rates of anxiety symptoms compared to those in community mental health treatment [23, 68]. For example, Rosenblatt and colleagues [23] found that youth involved in mental health services with no recent arrest record were approximately 3.5 times as likely to have an anxiety disorder compared to youth who utilize mental health services and have a recent arrest record. In addition, Garland and colleagues [68] compared rates of mental health disorders across service sectors and found that youth in mental health services, compared to youth in juvenile justice, had higher rates of anxiety disorder (JJ: 8.5%, MH: 11.9%), although the difference was not statistically significant.

Externalizing issues were examined by use of the Externalizing Scale which included items related to proactive and reactive aggression. In line with predictions, the relationship between case type and externalizing symptoms was moderated by sex. Therefore, case type differences within each sex were explored. It was found that males in the youth justice group scored lower in proactive and reactive aggression than male inpatients but they did not differ from male outpatients, and male inpatients scored significantly higher than outpatients. However, there was an opposite pattern in females- females in the youth justice group scored higher in proactive and reactive aggression than female outpatients but did not differ from female inpatients, and female inpatients scored higher in externalizing symptoms than outpatients. Notably, in line with hypothesis, the differences between males in the youth justice group and male patients were less pronounced than the differences between females in the youth justice group and female patients. The greatest difference in proactive and reactive aggression was found between female youth justice and female outpatients. Therefore, among females, females in the youth justice group endorsed more aggressive behaviours, as measured by the externalizing scale.

The results of the current study are consistent with other research that found females involved in the justice system scored highest in anger and irritability compared to males in the justice system, as well as males and females in the community [29]. Among detained adolescent females, those who scored very high in aggressive behaviour (“severely aggressive” group) were also more likely to have a diagnosis of ADHD, ODD, CD, or substance use concerns compared to females exhibiting less aggressive behaviour [69]. Therefore, there may be differentiated patterns of sex differences for aggression between youth who receive mental health services in inpatient or outpatient programs compared to youth in the justice system.

These results highlight a need for gender-specific interventions to address the specialized needs of females [70]. One gender-specific intervention, Girl’s Circle [71] is a strength-based group intervention using relational theory, skills training and resiliency to improve well-being. Areas of focus include body image, interpersonal relationships, and effectively expressing emotions. Results have indicated improved self-efficacy and a reduction in self-harm and substance use. With respect to next steps, policies and practices designed to address the unique needs of females within the youth justice system is needed, especially given that there are large gender gaps in opportunities for services as well as gender biases [72]. Additionally, future research is also required to determine the effectiveness and efficacy of gender-specific approaches to intervention, and any differential impact depending on specific factors (e.g., race, culture), both within and outside of the justice system.

### Limitations

The results and discussion of this article should be considered in light of several limitations. First, the current study did not discuss findings related to youth substance use, an important issue in the discussion of mental health and justice-involved youth. Related research is currently underway to examine these issues. Second, the current study compared three case types of youth. Youth each received a unique case record number and only their initial assessment was utilized within either the youth justice, inpatient or outpatient service sector. Other relevant literature indicates high levels of overlap between the use of inpatient and outpatient mental health services and involvement in the youth justice system. Consequently, it will be important for future research to examine cross-sectoral usage of mental health services to improve continuity of care, reduce assessment burden and facilitate integrated care plans for various service sectors (e.g., schools, mental health agencies, hospitals and youth justice facilities) as these youth are often not distinct and use services across multiple sectors. In addition, due to the cross-sectional nature of the data, causal inferences are not possible. Future research should investigate the longitudinal nature of mental health and related issues across service sectors.

Within the mental health facilities, the interRAI instrument was administered as part of standard of care; however, within the youth justice sample, the instrument was completed as part of a research study which may have had some impact on the results of the study (e.g., self-selected sample, less severe in terms of mental health need). For the youth justice sample, the assessment process was part of a pilot project and these individuals were not seeking mental health services, unlike those youth within the inpatient and outpatient sample. Consequently, the results could reflect an underestimate of the mental health needs of the youth justice sample.

Conversely, both inpatient and outpatient participants were referred for mental health services and received the assessment as part of their care. Future policy changes related to integrated health information systems designed to improved service system integration is needed given the number of youths who utilize multiple service sectors. This would allow for improved early identification, triaging and foster an evidence-based case finding methodology to improve evidence-based care [58].

It was noted that only 25% of those in youth justice participated. Low rates of voluntary research in the youth justice population have been found to be influenced by a number of factors, including distrust of researchers or institutional staff, gaining parental or guardian consent, and the transient nature of the population [73–75]. Additionally, rates of research participation have declined over the years [76]. To complicate things further, due to the vulnerable nature of incarcerated youth, a variety of additional ethical requirements were identified prior to youth participation (e.g., recommending legal advice prior to participation, concerns regarding disclosing information resulting in additional charges, stigma regarding mental health) which deterred the youth justice population from participating. As such, the low participation rate within the youth justice group is not entirely unexpected but does limit the generalizability of the results.

The current study also did not include various other factors that have been found in previous research to be related to mental health needs and involvement with in the youth justice system such as race, ethnicity and socioeconomic status due to specific regulations and ethical implications around specific vulnerable subpopulations. Previous research has found that youth who have been raised in low socioeconomic status or with limited access to resources are over-represented in the youth justice system [77, 78]. It is likely that these factors play an important part in the findings, resulting in differences across the three groups. Consequently, it is important to address both risk and protective factors associated with reducing the risk factors and increasing protective factors to foster resiliency in these high-risk youth. Responding to the mental health needs of vulnerable families as early as possible, utilizing early intervention in preschools/schools (e.g., children with issues related to readiness to learn, emotion regulation issues, learning difficulties) is likely to have the most benefit to circumvent the long-term sequelae related to youth in conflict with the law.

Finally, the study encountered a low rate of female participants in the youth justice group (23% identified as female). This rate directly reflects the nature of this population in Canada as previous studies have found that males outnumber females in youth justice, averaging approximately a 3:1 male to female ratio [79, 80]. Nonetheless, given the small sample of females within the youth justice sample, more detailed examination of within group differences was not possible (e.g., comparisons of females in youth justice who experienced parental abandonment compared to those who did not).

## Conclusions

Although a large number of studies have highlighted the high rates of mental health issues in youth justice populations, fewer have compared those rates between youth receiving mental health care both in the community and residentially. The current study has directly compared mental health needs across three service sectors: youth justice, inpatients, and outpatients, using the same assessment framework.

Of particular interest were the trauma-related characteristics of justice-involved youth. Although numerous studies have already highlighted the robust relationship between trauma exposure and justice involvement, few have directly compared trauma-related experiences between those in the youth justice system and those receiving mental health care, both residentially and in the community. The types of trauma experienced most commonly by justice-involved youth (e.g., parental abandonment, living in a violent neighbourhood) may represent barriers to accessing mental health supports and thus long-term lack of services and opportunity for early intervention.

Most importantly, the study highlighted important differences in the mental health needs of justice-involved youth, particularly when considering sex differences. Inpatient and youth justice samples appeared similar with respect to mental health needs. However, girls exhibited different mental health needs than boys, which has implications for gender-specific interventions to address the specialized needs of females.

## Data Availability

The dataset analyzed during the current study are not publicly available due to reasons of confidentiality and to protect the privacy of participants.

## Abbreviations

ANCOVA: Analysis of covariance
CAPs: Collaborative Actions Plans
CD: Conduct disorder
ChYMH: Child and Youth Mental Health
DICA-R: Diagnostic Interview for Children and Adolescents – Revised
DISC-IV: Diagnostic Interview Schedule for Children Version IV
DSM: Diagnostic and Statistical Manual of Mental Disorders
GLM: Generalized linear modelling
MAYSI-2: Massachusetts Youth Screening Instrument Version 2
ODD: Oppositional defiance disorder
PTSD: Posttraumatic stress disorder
YJ: Youth justice
YJCF: Youth Justice Custodial Facilities

## Footnotes

1 We probed for case type × sex interactions for traumatic life events. Although some of them were statistically significant, a decision was made to not report them due to very wide 95% confidence intervals for odds ratios. Instead, all binary logistic regression models control for sex and age differences.
